# Gut Mycobiome: Latest Findings and Current Knowledge Regarding Its Significance in Human Health and Disease

**DOI:** 10.3390/jof11050333

**Published:** 2025-04-22

**Authors:** Bogdan Severus Gaspar, Oana Alexandra Roşu, Robert-Mihai Enache, Monica Manciulea (Profir), Luciana Alexandra Pavelescu, Sanda Maria Creţoiu

**Affiliations:** 1 Department of Surgery, Carol Davila University of Medicine and Pharmacy, 050474 Bucharest, Romania; bogdan.gaspar@umfcd.ro; 2 Surgery Clinic, Bucharest Emergency Clinical Hospital, 014461 Bucharest, Romania; 3 Department of Morphological Sciences, Cell and Molecular Biology and Histology, Carol Davila University of Medicine and Pharmacy, 050474 Bucharest, Romania; oana-alexandra.rosu@rez.umfcd.ro (O.A.R.); monica.profir@rez.umfcd.ro (M.M.); luciana.pavelescu@umfcd.ro (L.A.P.); 4 Department of Oncology, Elias University Emergency Hospital, 011461 Bucharest, Romania; 5 Department of Radiology and Medical Imaging, Fundeni Clinical Institute, 022328 Bucharest, Romania; robert-mihai.enache@rez.umfcd.ro

**Keywords:** gut mycobiome, fungi, gut microbiota, dysbiosis

## Abstract

The gut mycobiome, the fungal component of the gut microbiota, plays a crucial role in health and disease. Although fungi represent a small fraction of the gut ecosystem, they influence immune responses, gut homeostasis, and disease progression. The mycobiome’s composition varies with age, diet, and host factors, and its imbalance has been linked to conditions such as inflammatory bowel disease (IBD) and metabolic disorders. Advances in sequencing have expanded our understanding of gut fungi, but challenges remain due to methodological limitations and high variability between individuals. Emerging therapeutic strategies, including antifungals, probiotics, fecal microbiota transplantation, and dietary interventions, show promise but require further study. This review highlights recent discoveries on the gut mycobiome, its interactions with bacteria, its role in disease, and potential clinical applications. A deeper understanding of fungal contributions to gut health will help develop targeted microbiome-based therapies.

## 1. Introduction

The human gastrointestinal tract is a complex ecosystem hosting a diverse array of microorganisms, including bacteria, viruses, and fungi. While bacterial communities have long been the primary focus of gut microbiome research, the fungal component, known as the mycobiome, has gained increasing attention for its crucial roles in health and disease [1].

Recent studies using amplicon sequencing and culture-based techniques suggest that the mycobiome may develop before birth [2]. However, this remains controversial, as other studies argue that the mycobiome emerges only after birth [3]. Despite these discrepancies, there is broad agreement that the gut mycobiome evolves significantly from infancy to adulthood, exhibiting greater variability and instability between individuals compared to the bacterial microbiota [4,5]. Multiple factors influence its composition throughout life, including maternal and infant diet, delivery mode, maternal age, geography, ethnicity, lifestyle, and dietary habits. For example, increased levels of *Saccharomyces cerevisiae* are observed in urban populations, while specific fungal species from the *Penicillium* and *Naumoyozyma* genera are more prevalent in Chinese individuals. Likewise, dietary patterns influence mycobiome composition, with *Candida* spp. flourishing in carbohydrate-rich diets and showing reduced abundance in protein-enriched diets [6,7,8,9].

Although fungi constitute only 0–1% of the gut microbiota, they have profound effects on host physiology and immunity [4]. Advances in deep sequencing and microbial cultivation have led to the identification of numerous fungal species, including keystone species involved in human health and disease [4,10]. The gut mycobiome interacts dynamically with bacteria and viruses, shaping health and disease states [4]. For instance, *Candida* spp. influence bacterial communities by competing for nutrients, producing antimicrobial peptides, and generating secondary metabolites [11,12]. Dysbiosis-driven increases in *Candida albicans* levels can negatively affect dominant gut bacteria in preterm infants and reduce the efficacy of fecal microbiota transplantation (FMT) [11,12]. Another key player is *Malassezia restricta*, which can activate the C3 complement cascade, driving gut inflammation and exacerbating IBD and pancreatic cancer [13,14,15]. Additionally, bacterial–fungal interactions shape microbiome stability, with bacteria modulating fungal germination and hyphal growth via fatty acid production [16,17]. Notably, *C. albicans* has been found to protect *Helicobacter pylori* from stomach acidity by sequestering it within its vacuoles, demonstrating interkingdom interactions that impact colonization and survival [18].

**Figure 1:**
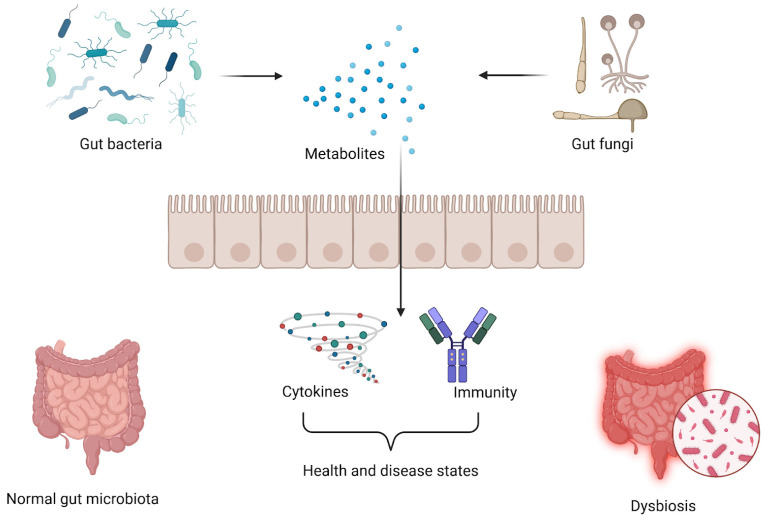
A balanced microbial environment and dysbiosis influence the interactions between bacteria and fungi within the gut microbiota. Under normal conditions, these microorganisms engage in cooperative relationships that support gut health. However, in dysbiosis, their interactions can become antagonistic, potentially disrupting immune regulation and contributing to disease development and progression. Created with Biorender.com (accessed on 16 February 2025).

In dysbiosis, the gut mycobiome becomes dominated by opportunistic fungal pathogens, potentially influencing immune responses and contributing to both intestinal and extra-intestinal diseases. These include inflammatory bowel disease (IBD), irritable bowel syndrome, celiac disease, metabolic disorders, neurological conditions, autoimmune diseases, and even cancer [14,19,20,21,22]. Dysbiosis can arise from bacterial or viral infections, antifungal treatments, or chronic alcohol consumption, with associated shifts in fungal composition, such as *C. albicans* overgrowth and bacterial microbiome alterations [12,23,24]. While the exact mechanisms remain incompletely understood, studies suggest that these interactions change over the disease course [4]. One potential mechanism is the activation of Th17 and IL-23-mediated immune responses by *C. albicans* cell wall components, leading to microbiota disruption and inflammation [11,25]. Increased *C. albicans* levels can trigger antifungal Th17 immune responses, which may reach the lungs and contribute to airway inflammation [26]. The interactions between bacteria and fungi within the gut microbiota are summarized in Figure 1.

Recent research has not only uncovered the diversity and functional significance of gut fungi but also highlighted their potential therapeutic applications [4]. Among the most researched strategies for modulating the gut mycobiome are dietary interventions, probiotics (both fungal and bacterial strains), antifungals, antibiotics, and fecal microbiota transplantation (FMT) [4]. For instance, coconut oil-rich diets can inhibit *C. albicans* overgrowth, while enteral nutrition in pediatric Crohn’s disease (CD) patients has been linked to reduced *C. albicans* abundance [27,28]. Probiotics, particularly bacterial and fungal strains, can produce antimicrobial compounds that limit fungal colonization, combat infections, and treat various diseases, such as *Clostridium difficile* infection (CDI) and inflammatory bowel disease [4]. Antifungal therapies, such as fluconazole, have been effective in reducing *C. albicans* colonization in ulcerative colitis (UC) but may disrupt microbiota balance in prolonged use [4,29]. Notably, antibiotics can have a more enduring impact on the gut fungal community than on bacterial populations [30]. Lastly, FMT has shown promise in modulating fungal overgrowth, particularly in UC (in the case of increased *Candida* spp. abundance in recipients before FMT and reduced *Candida* spp. levels after FMT), though its effectiveness may be compromised by pre-existing fungal dysbiosis [31,32].

This narrative review aims to synthesize current knowledge on the gut mycobiome, emphasizing its development, interactions with bacterial microbiota, role in health and disease, and potential therapeutic interventions.

## 2. Gut Mycobiome: Important Fungi Residing in the Human Gut

The human gut mycobiome is mainly composed of yeast species, with the most commonly identified genera being *Candida*, *Saccharomyces*, and *Malassezia* [33,34]. However, the relative abundance of these genera can vary significantly amongst individuals, being strongly influenced by factors such as diet, geography, antimicrobial use, and health status [34,35].

**Figure 2:**
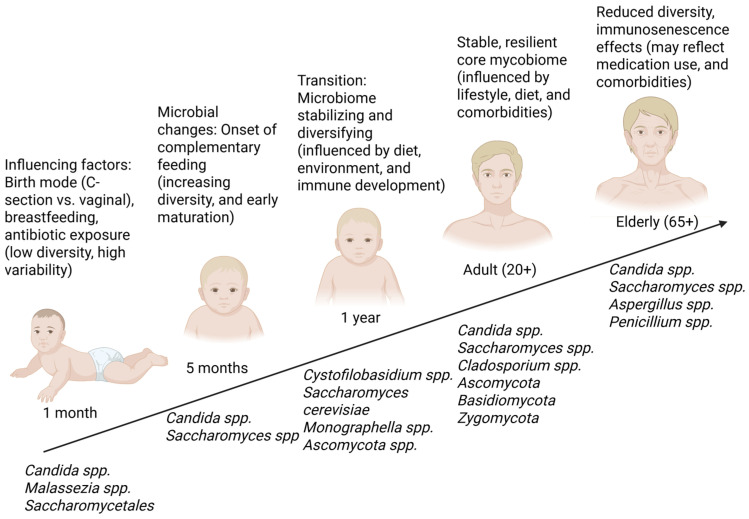
Updated dynamics of early-life gut fungal colonization. This figure illustrates the revised pattern of gut fungal colonization in infants, integrating longitudinal data from the recent studies. The updated data indicate that initial colonization occurs more rapidly than previously reported, with significant shifts in fungal diversity observed following dietary transitions and environmental exposures during the first year of life. These findings redefine our understanding of the establishment and maturation of the gut mycobiome compared to earlier studies. Created with Biorender.com (accessed on 20 April 2025).

The composition of the gut mycobiome varies with age [4]. Recent longitudinal studies have significantly advanced our understanding of gut mycobiota colonization dynamics in early life and beyond [36]. Unlike earlier studies, which primarily reported a gradual increase in fungal diversity starting from infancy, the updated data reveal that colonization begins robustly during the first month and is rapidly influenced by factors such as the mode of delivery (vertical transmission), feeding practices (breastfeeding vs. formula), and environmental exposures [36]. During the first month of life, the gut mycobiome is predominantly composed of fungal species from the phyla *Candida* spp., Ascomycota (*Saccharomycetales* order), and Basidiomycota (*Malasseziales* order), with *Malasseziales* experiencing a significant decline by five months of age. By 5 months, fungal diversity increases (gradual increase in fungal diversity, particularly within the *Saccharomycetales* order), shaped by environmental exposure and dietary transition (solid foods and other dietary changes) [36,37]. These updated findings not only refine the timeline of mycobiome maturation but also underscore the impact of early-life interventions on long-term gut health [36]. As infants begin consuming a more diverse diet, the dominant fungal genera in the gut shift to include *Saccharomyces cerevisiae*, *Cystofilobasidium* spp., *Ascomycota* spp., and *Monographella* spp., reflecting the influence of solid food introduction and microbial maturation [36,38]. Over the following years, the gut mycobiome gradually transitions to resemble the adult gut mycobiome, which is characterized by the presence of *Ascomycota*, *Basidiomycota*, and *Zygomycota*, with *Candida*, *Saccharomyces*, and *Cladosporium* being the most abundant genera in healthy adults [39]. Fungal composition in adulthood is shaped by diet, lifestyle, immune status, and host genetics [36]. In elderly populations, changes in immunity, diet, and medication use may contribute to shifts in fungal composition, with an increased prevalence of *Penicillium*, *Candida*, *Saccharomyces*, and *Aspergillus* becoming more prevalent [36,40]. The changes in gut mycobiome composition throughout life are illustrated in Figure 2.

Recent longitudinal studies using TEDDY metagenomic libraries have provided valuable insights into early-life gut mycobiome dynamics and their potential implications for autoimmunity. In particular, Auchtung et al. demonstrated that children at risk for type 1 diabetes exhibit specific mycobiome signatures before clinical onset [36]. The conclusions of this work emphasize that early fungal colonization patterns—shaped by factors such as delivery mode, feeding practices, and environmental exposures—may serve as potential biomarkers for subsequent autoimmune risk. Moreover, integrating these findings with recent insights into the mechanistic roles of fungal components in immune regulation and cross-kingdom metabolic crosstalk extends our understanding of how gut fungi might actively influence disease development, as also discussed in broader reviews [41,42,43]. These recent advances not only refine our picture of the temporal dynamics of mycobiome maturation in infancy but also provide a strong rationale for further investigating the role of early-life fungal dysbiosis in the etiology of type 1 diabetes.

A comprehensive study by Hallen et al. identified the genera *Cutaneotrichospon*, *Saccharomyces*, *Aspergillus*, *Candida*, and *Malassezia* to be the most prevalent fungal genera in fecal samples from healthy human gut microbiota [39]. These findings align with previous research by Nash et al., which also identified *Saccharomyces*, *Candida*, and *Cladosporium* as dominant fungal genera in the human gut [5].

*Candida* species are commensal in the human gut but can become pathogenic under specific conditions such as antibiotic use, immunosuppression, and gut dysbiosis [44,45]. *C. albicans*, *C. glabrata*, *C. tropicalis*, *C. parapsilosis*, and *C. lusitaniae* are the species of *Candida* most frequently associated with infection and inflammation [46]. The excessive use of broad-spectrum antibiotics profoundly impacts fungi residing in the human gut, particularly *C. albicans*, leading to overgrowth and antibiotic-associated diarrhea [47]. A study by Sokol et al. identified elevated levels of *Candida* in patients with gastrointestinal disorders such as CD and UC [48]. *C. albicans* facilitates mucosal invasion by adhering to the intestinal epithelial cells and producing virulence factors [49]. In a healthy gut, *Candida* is typically kept in check by *Lactobacillus* species, which produce antimicrobial compounds that inhibit its overgrowth [50]. In addition, colonocytes play a role in bringing *C. albicans* back to their commensal state by signaling immune cells and activating IL-22, which stimulates the production of secretory IgA and β-defensins [34]. Charlet et al. found that in mice with induced colitis, elevated *C. glabrata* levels correlated with increased inflammation and mortality, primarily due to higher chitin levels in the fungal cell wall during colonization. Conversely, a deficiency in chitin synthase-3 was linked to reduced inflammatory markers [51]. Elevated levels of *Candida tropicalis* have been directly linked to heightened inflammation in CD in humans and colitis in mice. This occurs through mucin-degrading bacterial modifications, tight junction protein expression alterations, and increased gut permeability [52,53]. A study by Sun et al. demonstrated that *Candida parapsilosis* can induce diet-related obesity in fungi-free mice by increasing fungal lipase production, leading to elevated free fatty acids in the gut. This highlights the potential of targeting this fungus in obesity treatment strategies [54]. *Clavispora lusitaniae* (formerly *Candida lusitaniae*) primarily affects immunocompromised patients, such as those with hematological malignancies, causing severe infections in the digestive and urinary systems. Additionally, it has been associated with resistance to certain antifungal agents, including amphotericin B [55]. Another *Candida* species, *Geotrichum candidum*, was identified in a study by Noor-UI et al. as a potential probiotic for fish, demonstrating benefits such as enhanced feed utilization, improved immunity, and reduced disease resistance [56].

The genus *Saccharomyces* includes several species, with *S. cerevisiae* being the most well-known. *S. cerevisiae* is a common yeast found in dietary sources such as fermented foods and bread [57]. *S. cerviaseae* var. *boulardii* (*S. boulardii*) is a probiotic strain that has been shown to modulate the gut immune response, enhance the integrity of the gut barrier, and protect against traveler’s diarrhea and CDI [58,59]. According to several studies, *S. boulardii* has also been shown to have antineoplastic effects. One study by Chen et al. showed that *S. boulardii* can inactivate the EGFR pathways, reduces cell proliferation, and promotes apoptosis. In addition, it was shown to reduce intestinal tumor growth and dysplasia in mice with colon cancer [60].

*Malassezia* is traditionally considered a skin-dwelling fungus associated with several skin conditions, such as dermatitis and pityriasis. It is frequently identified in several other environments, including the murine and human gut [13]. Due to technological advances, specifically NGS studies, several publications have reported the presence of *Malassezia* in healthy gut samples in recent years, confirming that it is part of the gut microbiota [61,62,63,64]. *Malassezia* has been associated with several diseases, including inflammatory bowel disease, colonic polyps, intestinal cancer, pancreatic cancer, and Alzheimer’s disease [14,15,48,65,66]. The role of *Malassezia* in developing inflammatory bowel disease has been characterized by Sokol et al. Limon et al., who suggested that *Malassezia* might exacerbate inflammation in genetically susceptible individuals [15,48]. Several researchers have demonstrated the role of the gut microbiota in the development of colorectal cancer (CRC); however, the implications of *Malassezia* strains have only recently been demonstrated by Gao et al. and Coker et al. [65,66,67]. How *Malassezia* can lead to the development of CRC remains unclear. However, *Malassezia* species have been associated with inflammatory responses that could lead to DNA damage and tumorigenesis [68]. In addition, it can produce potent aryl hydrocarbon receptor (AhR) ligands, which may promote tumor development by modifying UV radiation carcinogenesis, altering cell cycle progression and inhibiting apoptosis [69].

*Aspergillus* and *Penicillium* are common environmental molds that can colonize the human gut primarily through dietary intake. Although they are considered transient members of the gut mycobiome, certain species within these genera produce mycotoxins that have important health consequences. *Aspergillus species*, particularly *A. flavus* and *A. parasiticus*, produce alfatoxins, which are hepatotoxins that have been associated with hepatocellular carcinoma [70]. These *Aspergillus* species are frequently found in peanuts or peanut butter, and their growth is favored by warm, humid weather [71]. Similarly, *Penicillium* species, such as *P. verrucosum* and *P. nordicum*, produce ochratoxin A, a nephrotoxic compound associated with kidney damage and an increased risk of renal disease, including the development of urothelial carcinoma. Ochratoxin A contamination has been reported in products like cereals, coffee, and dried fruits [72]. Table 1 synthesizes the most important key findings regarding the effects of different fungal genera on human health and disease.

## 3. The Balance Between Gut Fungi and Bacteria in Healthy Subjects

In healthy people, bacteria and fungi live in a delicate equilibrium, fighting for resources and available space while occasionally interacting favorably. Preventing bacterial overgrowth and preserving gut homeostasis rely on this balance [73].

Certain bacteria and fungi interact synergistically to support gut health. For example, some bacterial species produce biofilms that help stabilize fungal populations, while fungi contribute to fiber degradation, supporting bacterial fermentation [73,74]. For instance, fungi like *Candida* can improve their colonization by using the biofilms produced by specific gut bacteria [75,76].

A balanced gut microbiome helps regulate digestion, nutrient absorption, and immune function [77]. This harmony reduces the risk of gastrointestinal disturbances, infections, and chronic inflammation, supporting overall metabolic and immune health [78,79].

Regarding the role of bacterial metabolites in fungal regulation inside the gut microbiota of healthy individuals, many aspects of SCFAs (short-chain fatty acids) can be emphasized [80,81]. SCFAs such as butyrate, propionate, and acetate, produced by the bacterial fermentation of dietary fibers, play a critical role in regulating gut fungi. These metabolites help inhibit pathogenic fungi, maintain gut pH, and strengthen the intestinal barrier. By modifying immune responses, SCFAs promote immunological tolerance and lower systemic inflammation [82,83]. Furthermore, regarding the systemic health benefits of SCFAs, there is evidence supporting the influence SCFAs have on energy metabolism, insulin sensitivity, and lipid regulation, contributing to a healthy weight and reducing the risk of metabolic disease [84,85]. SCFAs can influence the gut–brain axis, supporting cognitive function, mood regulation, and stress resilience in healthy individuals [86,87]. Another positive aspect of gut microbiota metabolites is that butyrate strengthens the gut lining, preventing leaky gut and systemic inflammation [88,89].

The immune system is influenced and trained by the gut microbiota. The equilibrium between bacteria and fungus in healthy people guarantees the right immune responses, avoiding infections and over-inflammatory reactions [90]. Regulatory T cells (Tregs), which support immunological tolerance and avoid needless immune activation against innocuous microorganisms and environmental antigens, are developed by commensal bacteria [91]. Although TH17 cells play a crucial role in defense against fungal infections, their activity is strictly controlled in healthy people to avoid excessive inflammation, which may result in autoimmune problems [92]. A healthy immune system promotes general health and infection resistance by lowering the likelihood of allergies, autoimmunity, and chronic inflammatory diseases [93].

Although antibiotics are necessary for the treatment of bacterial illnesses, their usage may unintentionally alter the gut microbiota, which can cause fungal overgrowth in otherwise healthy people [94]. Temporary problems like bloating, diarrhea, or yeast infections may result from this imbalance. Over time, the gut microbiota of healthy people usually recovers from antibiotic-induced disturbances, especially when supportive measures like probiotic consumption and a diet high in fiber are taken [95]. Probiotic and prebiotic use, along with thoughtful antibiotic use, are some preventative measures for healthy individuals [96]. Limiting needless antibiotics helps preserve microbial diversity and resilience while introducing beneficial bacteria and encouraging their growth can assist in restoring equilibrium following an infection or antibiotic use [94,97].

By seeing direct interactions between particular bacterial and fungal species, co-culture experiments enable researchers to pinpoint the mechanisms that maintain microbial balance in healthy people [98]. The intricate ecosystem of the human gut is replicated by sophisticated in vitro models, which shed light on the interactions between bacteria and fungi to preserve health [99]. Studying the effects of particular bacteria and fungi on health in a controlled setting is made possible by germ-free mice, which have no microbiota [99]. Mice that have been colonized with the microbiota of healthy humans can reveal information about how the gut microbiota supports a range of physiological functions, from immune regulation and brain function to digestion and metabolism [100,101,102,103].

By using sequencing methods, the gut microbiome may be thoroughly examined, and patterns of bacterial and fungal diversity linked to health can be found [104].

These techniques provide a better understanding of the function that microbial communities play in preserving health by illuminating how they affect host gene expression and metabolite synthesis [105,106]. By identifying individual differences in microbiota composition, multi-omics techniques can inform individualized dietary and lifestyle recommendations to maximize health [107].

## 4. Gut Mycobiome Characterization in Diseases

The interaction of fungi, bacteria, the host immune system, and environmental factors influences a wide range of diseases, from metabolic, autoimmune, infectious, and neurological conditions to gastrointestinal disorders [4,108].

The gut mycobiome, which is the fungal component of the gut microbiota, constitutes approximately 0.1% of the total microbiome. Despite its small proportion, it plays a crucial role in maintaining gut homeostasis and is implicated in various pathological conditions [34,109].

Bacterial and fungal populations are dysbiotic in CD and UC, two chronic inflammatory gastrointestinal disorders [110]. Usually tied to skin disorders, *Malassezia* species have been discovered in CD patients’ guts and are connected to inflammation by interacting with immune receptors, including CARD9 [15,111]. Patients with IBD are frequently dominant in *C. albicans*. It can exacerbate inflammation by penetrating the mucosal barrier in its hyphal form [112,113,114].

Regarding intestinal fungi, antifungal secretory immunoglobulin A, and the mechanisms of pathogenesis in CD, studies show that disease activity is correlated with decreased fungal diversity and an increased abundance of pathogenic fungi, particularly *Candida* [115,116,117].

By forming biofilms with bacteria, fungi increase resistance to antimicrobial therapies and the immune system [118]. TH17-mediated inflammation is promoted by fungi’s β-glucans, which activate pattern recognition receptors (PRRs) like Dectin-1 [119].

The symptoms of IBS, a functional gastrointestinal illness, include bloating, changed bowel patterns, and abdominal discomfort [120]. In addition to altered fungal–bacterial ratios that might affect gut motility and immunological responses, studies have shown elevated *Candida* spp. in IBS patients, which may be a contributing factor to bloating and visceral hypersensitivity [121,122].

The enteric nervous system may be impacted by fungal metabolites, which might lead to symptoms including pain and discomfort [123]. Fungal dysbiosis-induced inflammation may make IBS symptoms worse [124].

Through intricate relationships between bacterial populations and host metabolism, the mycobiome plays a part in the gut microbiome’s growing recognition of its function in metabolic control [4]. Research has indicated that obese people have higher levels of *Candida* species and *Saccharomyces cerevisiae*, *Mucor*, and *Rhodotorula*, while having a lower variety of helpful fungus [125]. Insulin resistance has been associated with altered fungal profiles, such as increased levels of *Candida* and lower levels of helpful fungi like *Pichia* [126]. Due to their ability to trigger the immune system and/or generate toxic metabolites, *C. albicans*, *Aspergillus*, and *Meyerozyma* may be potential pathogens of metabolic disorders [125]. The overgrowth of fungi can damage the gut and cause systemic inflammation, which is a major contributing factor to metabolic syndrome [125]. SCFA synthesis, which is essential for controlling glucose and lipid metabolism, can be interfered with by fungal dysbiosis [127].

The systemic autoimmune illness known as rheumatoid arthritis (RA) is defined by persistent joint inflammation. A typical feature of this autoimmune disease is fungal dysbiosis [128]. *C. albicans* levels are elevated in RA patients, which may precipitate or worsen autoimmune reactions [129]. It has been noted that commensal fungi such as *Saccharomyces* spp. have become less diverse [130]. The mechanisms of fungal involvement, in this case, include immune modulation, where fungal components can activate dendritic cells and Th17 pathways, contributing to systemic inflammation, and molecular mimicry, where fungal antigens may resemble host tissues, triggering autoimmune responses [131,132].

Mycobiome changes are seen in multiple sclerosis (MS) or encephalomyelitis, other inflammatory diseases marked by the demyelination of neurons in the central nervous system [133]. Neuroinflammation may be exacerbated by the changed gut microbiomes of MS patients, which show higher levels of *Candida* and *Saccharomyces* spp. [134]. Two possible explanations include the gut–brain axis, which may mediate between immune signaling and fungal metabolites that impact neuroinflammatory pathways, and the fungal activation of Th17, which may worsen central nervous system inflammation in MS [135,136,137]. Airway inflammation and systemic allergy reactions may be impacted by fungal dysbiosis in the gut. Increased allergic reactions and asthma exacerbations have been linked to *Aspergillus* and *Candida* species [138,139,140]. Immune skewing, in which fungal dysbiosis may enhance Th2-mediated immune responses, thereby contributing to allergic sensitization or the potential for the gut mycobiome to impact lung immunity through the systemic circulation of immune modulators and microbial metabolites via a gut–lung axis, is the mechanism underlying these processes [141].

Opportunistic fungal infections are more common in those with weakened immune systems, such as those receiving chemotherapy or living with HIV [142,143]. Under immunocompromised conditions, opportunistic fungi like *Candida* may spread from the gut to other parts of the body [143,144]. Interactions between kingdoms can increase virulence and biofilm formation, making treatment more difficult and increasing the risk of recurring infections [145]. Although fungal interactions can affect the severity of the condition, CDI is a serious bacterial infection that is frequently brought on by the use of antibiotics [146,147]. Fungal overgrowth, especially that of *Candida* spp., can result from antibiotic-induced bacterial depletion, worsening intestinal inflammation and hindering healing. Patients with CDI may benefit from bacterial treatments combined with mycobiome modification [146,148,149,150].

The gut mycobiome may impact mood and mental health through metabolite formation and immunological regulation. Symptoms of schizophrenia, anxiety, and depression have been connected to changes in fungus variety and abundance [151]. By producing cytokines, fungal dysbiosis may exacerbate systemic inflammation, which is linked to depression [151]. Moreover, neurotransmitter production may be impacted by fungal interactions with bacteria that produce SCFAs [152].

The development and progression of CRC are influenced by the gut microbiome, which includes the mycobiome. In addition to fewer helpful fungus, CRC patients have been reported to have higher levels of *C. albicans* and *Malassezia* spp. [153,154]. While fungal metabolites can affect the proliferation and death of cancer cells, fungal-induced inflammation may also produce an environment that is conducive to tumorigenesis [155]. Chemotherapy and radiation therapy for cancer can alter the gut microbiota, which can result in fungal overgrowth and related problems. Immunosuppression makes people more vulnerable to invasive fungal infections, which makes cancer treatment results more difficult [156,157]. By controlling fungal dysbiosis using antifungal medications and microbiome modification, therapy results and consequences may be enhanced [158].

**Figure 3:**
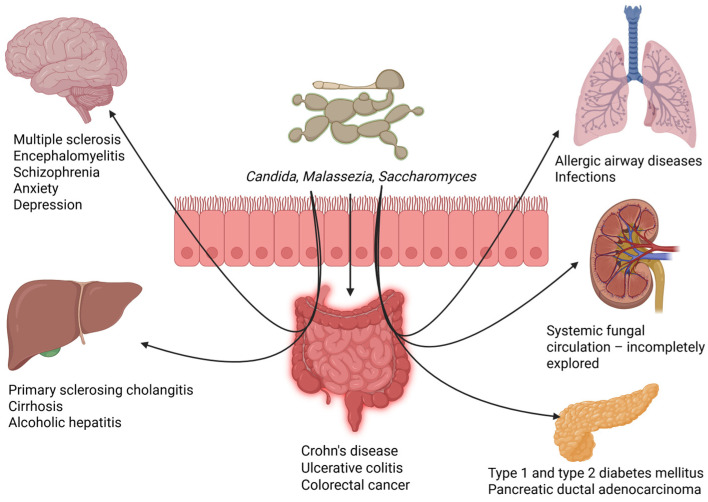
The relationship between the gut mycobiome and the main affected organs, highlighting the gut–brain, gut–lung, gut–liver, gut–kidney, and gut–pancreas axes. At the center of the image, the intestine is depicted as the primary reservoir of fungi, including species such as *Candida*, *Malassezia*, and *Saccharomyces*. Arrows indicate interactions between the gut and different organs. Created with Biorender.com (accessed on 20 April 2025).

The gut mycobiome plays a significant role in liver disease through the gut–liver axis. Studies have shown that patients with primary sclerosing cholangitis, cirrhosis, and alcoholic hepatitis exhibit increased mycobiome diversity, suggesting a link between fungal dysbiosis and liver pathology [159,160,161]. Conversely, kefir has been identified as a potential therapeutic agent for alcoholic fatty liver disease by modulating the gut mycobiome [162]. These findings highlight the therapeutic potential of targeting the gut–liver axis in liver disease management [163]. Similarly, the gut–kidney axis has been implicated in fungal infections. Research in mice with *Candida*-disseminated infections has shown that the kidneys carry the highest fungal burden, emphasizing their role in systemic fungal circulation. However, the exact contribution of gut mycobiome imbalance to renal disease pathogenesis remains largely unexplored [164]. The interplay between the gut mycobiome and the pancreas has also been observed in metabolic and oncological conditions. Patients with type 1 and type 2 diabetes mellitus exhibit elevated *C. albicans* levels, suggesting a potential role of fungal dysbiosis in diabetes development [165]. Additionally, increased *Malassezia* spp. levels have been found in patients with pancreatic ductal adenocarcinoma, indicating a possible gut–pancreas connection via the sphincter of Oddi [14,166]. However, further research is needed to determine whether gut mycobiome dysbiosis is a driving factor in oncogenic progression or a consequence of the disease. Understanding these gut–organ axes may open new avenues for targeted fungal-based therapies in various diseases [163]. The relationship between the gut mycobiome and the main affected organs is illustrated in Figure 3.

A biofilm is a structured microbial community composed of bacteria and fungi encased in a self-produced polymeric matrix that adheres to surfaces. These biofilms can persist, reproduce, and influence human health and disease states [118]. Pathological biofilm formation is characterized by uncontrolled microbial growth, leading to infections and accounting for approximately 70% of microbial infections. These include periodontitis, chronic prostatitis, cystic fibrosis, endocarditis, and otitis media. Additionally, biofilm formation can lead to the contamination of medical devices such as prosthetic heart valves, central venous catheters, contact lenses, intrauterine devices, dental unit waterlines, and urinary catheters [167,168,169,170,171,172]. The pathogenicity of biofilms is driven by bacterial cell detachment, endotoxin production, immune evasion, and the development of a protective barrier [118,173,174]. While bacterial biofilms are well studied, fungal biofilms, particularly those involving *Candida* spp., play a significant role in infections and antimicrobial resistance. *Candida* biofilms exhibit high tolerance to antifungal treatments and can form mixed-species biofilms with bacteria, enhancing resistance and persistence [175,176]. Additionally, bacterial biofilms can impact the gut mycobiome, contributing to antimicrobial resistance against antibiotics and disinfectants [177]. However, studies suggest this resistance is lowest during the early biofilm formation stages, where antibiotic treatments and dietary supplements are most effective [178]. Studies have shown that the response to treatment also varies across mycobacterial species due to differences in metabolic states and resistance genes [118,179]. Increased antibiotic use has led to the emergence of resistant fungal biofilms, necessitating novel eradication strategies [175,176]. Approaches include surface modifications to prevent bacterial adhesion, probiotics (*E. faecium* and *Bifidobacterium adolescentis*), exopolysaccharide antagonists (e.g., N-acetylcysteine and benzimidazole), peptide-based vaccines, monoclonal antibodies, and mechanical removal methods like ultrasound or thermal shock therapy [118,180,181,182,183]. Given biofilms’ affinity for moist, nutrient-rich surfaces, enhanced preoperative and postoperative precautions and innovative biofilm-targeting therapies are crucial in combating fungal and mixed-species biofilm-associated infections [184].

## 5. Clinical Perspectives on the Possibilities for Calibrating the Gut Mycobiome

### 5.1. Antifungal Therapies

The overgrowth of fungi, especially opportunistic species like *C. albicans*, can lead to inflammation, systemic immunological activation, and gut dysbiosis [185]. Antifungal treatments aim to balance the gut microbiota and lessen fungal overgrowth. Antifungal drugs come in a variety of forms with a range of clinical uses [186]. Fungal cell death results from the azoles (fluconazole, itraconazole, and voriconazole) inhibiting the synthesis of ergosterol, a crucial component of fungal cell membranes [187]. Patients with severe *Candida* overgrowth or systemic fungal infections are treated with them [188]. For instance, fluconazole has been used to lessen inflammation in patients with fungal dysbiosis who have CD [189]. Azole use has many drawbacks, including the potential to change bacterial populations and exacerbate dysbiosis, as well as the risk of resistance if taken accordingly with the prescription method [190].

Polyenes, which bind to ergosterol and damage fungal cell membranes, are another class of antifungal agents. Examples of these include nystatin and amphotericin B [191,192]. Amphotericin B is used for severe systemic fungal infections but rarely for gut dysbiosis [125]. Nystatin is a non-absorbable antifungal utilized for gastrointestinal *Candida* overgrowth [193]. Nystatin has been used to treat small intestine fungal overgrowth (SIFO) and IBS to alleviate symptoms in patients suffering from fungal dysbiosis [194]. Conversely, polyenes may induce local irritation and be harsh on the intestinal lining [195,196]. Echinocandins (caspofungin and micafungin) target β-glucans to inhibit the formation of fungal cell walls [197]. They may help immunocompromised people with gut fungal overgrowth and are used for systemic fungal infections but not frequently for gut dysbiosis [198,199]. A study by Puškárová et al. investigating the antibacterial and antifungal properties of six essential oils (oregano, thyme, clove, lavender, clary sage, and arborvitae) found that they effectively inhibit mycelial growth. Their antifungal effects, whether fungicidal or fungistatic, varied depending on the fungal strain (*Cladosporium cladosporoides*, *Alternaria alternata*, *Aspergillus fumigatus*, *Chaetomium globosum*, and *Penicillium chrysogenum*). Additionally, the study revealed that the vapor administration of these essential oils was more effective than liquid application [200]. Research indicates that berberine possesses significant antifungal activity, notably against *C. albicans*. In murine models, berberine administration has been shown to reduce intestinal damage associated with fungal overgrowth and improve the balance of gut microbiota, thereby preventing dysbiosis [201]. An in vitro study conducted by Tonoue et al. examined the antimicrobial effects of Arctostaphylos uva-ursi (*A. uva-ursi*), a plant commonly used for urinary tract infections, on *Aspergillus flavus*, *Aspergillus fumigatus*, *Aspergillus niger*, *C. albicans*, and *Escherichia coli*. The results revealed that *A. uva-ursi* exhibited antimicrobial activity only against *C*. *albicans* and *E. coli*, with a stronger effect observed when using the herbal extract than the homeopathic mother tincture. However, the authors noted that their findings were inconclusive due to study limitations, including the absence of comparable research, the lack of isolation of the plant’s active ingredients, and variability in the extracts’ sources [202]. Additionally, a review by Gupta et al. emphasized the significant antibiotic properties of *A. uva-ursi* in treating urinary tract infections, primarily attributed to its glycoside arbutin, which is converted into hydroquinone. The authors highlighted arbutin’s multiple potential effects, including antiseptic, antibiotic, astringent, and diuretic properties, while calling for further research to better understand its therapeutic potential [203]. A study by Shi et al. investigated the effects of undecylenic acid on *C. albicans* skin infections and found that it serves as an effective antifungal agent. The study demonstrated that undecylenic acid inhibits *C. albicans* biofilm formation and prevents its transition from yeast to filamentous form, even at low treatment doses [204].

Although using antifungal drugs has many benefits, there are several drawbacks to these treatments as well. Although they lessen the fungal load, antifungals do not always return the microbial equilibrium [186,205]. Among other things, removing fungus without bacterial modulation can result in bacterial overgrowth, change gut function, and select for resistant fungal strains [158,206]. The most crucial takeaway is that fungal overgrowth could return without sustained microbiome support.

In cases of severe fungal dysbiosis, immunocompromised patients, or refractory IBD, IBS, or SIFO, antifungal medication should be evaluated on an individual basis [207,208]. Targeted antifungals that do not harm beneficial fungi should be the subject of future study.

### 5.2. Probiotics

Some bacteria compete with fungi for nutrition and space and have inherent antifungal qualities. The organic acids produced by *Lactobacillus rhamnosus* GG inhibit *Candida species* [209,210]. Reuterin is an antibacterial substance that works well against fungus and is produced by *Lactobacillus reuteri* [177,211,212]. *Candida* and *Bifidobacterium breve* compete for gut cell attachment sites [213,214]. *L. rhamnosus* supplementation decreased *Candida* colonization and enhanced gut barrier function in IBD patients [215,216,217]. Probiotics made from yeast such as *Saccharomyces boulardii* compete with *Candida* for resources, decreasing the creation of fungal biofilms and increasing the synthesis of IgA (which targets fungal infections); this non-pathogenic yeast stops fungal overgrowth [58,218,219]. It has been demonstrated that *S. boulardii* lowers the risk of fungal translocation in critically ill patients and is used to treat antibiotic-associated diarrhea [220]. One disadvantage of probiotics is that not all of them have antifungal qualities. Certain probiotics might not withstand stomach acid if they are not properly prepared [120]. Additionally, the overuse of probiotics in immunocompromised people may result in bacterial or fungal overgrowth. Supplementing with probiotics has potential as an adjuvant treatment, especially for fungal-associated dysbiosis, IBD, and IBS [221]. For the best results, strain selection and patient-specific characteristics must be taken into account.

### 5.3. Fecal Microbiota Transplantation

The goal of FMT is to restore gut microbial diversity, including fungal communities, by transferring stool from a healthy donor into a recipient [222]. Several findings about the function of FMT in mycobiome modulation indicate that it may be a focus for future research with promising outcomes in certain situations [223]. Following antibiotic treatment, FMT has been demonstrated to prevent excessive *Candida* overgrowth in CDI patients by restoring fungal equilibrium [146]. FMT has been proven to enhance illness outcomes and reduce fungal burden in patients with IBD and *Candida*-associated dysbiosis [224]. FMT may improve metabolic indicators in patients with metabolic syndrome by assisting in the recalibration of bacterial and fungal communities [225,226].

Fungal composition differs among donors, which causes variable results when it comes to the issues of FMT [227]. Over time, the transplanted mycobiome might change. There is still concern about unintentional pathogen transmission [228].

For gut dysbiosis, including fungal imbalances, FMT is a promising treatment [229]. However, before it can be widely used in conditions linked to fungi, standardization and safety procedures need to be improved.

### 5.4. Dietary Support

Diet has a major impact on the overall composition of the gut microbiome, which includes fungus [230]. A varied and balanced microbiome is supported in healthy people by diets high in fiber, whole grains, fruits, and vegetables [231]. Dietary fibers encourage the development of good bacteria that generate SCFAs, which support gut health and fungal population control [232]. Berries, green tea, and dark chocolate are examples of foods high in polyphenols that naturally have antifungal qualities that help maintain the delicate balance between bacteria and fungi [233]. Specific food patterns can also support a healthy microbiome [4,234]. The Mediterranean diet, which emphasizes fresh fruits, vegetables, and seafood, is, on the one hand, linked to increased microbial diversity and decreased systemic inflammation [235]. Conversely, a plant-based diet devoid of meat supports immunological and metabolic health by encouraging the diversity of beneficial bacteria and fungi [235]. Metabolic outcomes may be enhanced by dietary therapies that target both bacterial and fungal components of the microbiome [236]. Prebiotics and probiotics that promote fungal equilibrium may help control type 2 diabetes (T2D) and obesity [237].

Through microbial interactions and nutritional availability, diet has a significant impact on the gut microbiome, influencing fungal populations [238]. There are various strategies for dietary intervention to calibrate the mycobiome [239]. Restricting the intake of sugar and processed carbs can be the first step. Consuming too much sugar encourages the growth of fungi, especially *Candida* [240,241]. Patients with recurring infections have been treated with low-carb or ketogenic diets to lessen their fungal burden [242]. The daily consumption of meals high in fiber can be another strategy [243,244]. Beta-glucans, resistant starch, and inulin are examples of prebiotics that support the diversity of good microorganisms, including antifungal bacteria [79,245]. For instance, the Mediterranean diet, which is customarily high in fiber and polyphenols, promotes a balance of gut fungi and inhibits the growth of *Candida* [244,246]. Dietary modification is a foundational approach to gut mycobiome regulation, with high-fiber, low-sugar, and polyphenol-rich diets showing the most promise [239].

A comprehensive strategy that incorporates targeted antifungal medication, probiotic supplementation, FMT, and dietary interventions is needed to calibrate the gut mycobiome [34]. Antifungal medications offer short-term respite, but probiotics and dietary changes are the best ways to maintain mycobiome stability over the long run [34]. Personalized mycobiome modification techniques catered to specific medical problems should be the main emphasis of future research.

Since refined carbohydrates and rapidly digestible starches are the main energy sources for fungal development, limiting them is one of the most important dietary strategies for controlling fungal overgrowth [247].

For fungi and opportunistic bacteria, refined carbohydrates and quickly digested starches offer a readily available energy source [248]. In the digestive tract, simple sugars and quickly digested starches—such as those in white rice, pasta, potatoes, and processed grains—are hydrolyzed into glucose very fast [249]. This quick conversion raises blood sugar levels, which might upset the microbial balance and encourage fungal development [250].

Another facet of this subject is that prebiotic fibers are better for beneficial bacteria like *Lactobacillus* and *Bifidobacterium* than simple carbohydrates [251]. The overconsumption of carbohydrates can exacerbate intestinal dysbiosis by favoring harmful bacteria and yeast [252].

In addition, these carbohydrates contribute to intestinal disruption. The intestinal barrier integrity may be altered by the overabundance of fungal species, especially *C. albicans*, which increases the generation of mycotoxins and inflammatory chemicals [253,254]. Often called leaky gut syndrome, this illness is linked to systemic health problems, immunological dysregulation, and persistent inflammation [255].

An additional element of this subject is that prebiotic fibers, as opposed to simple carbohydrates, are what beneficial bacteria like *Lactobacillus* and *Bifidobacterium* thrive on [256]. Consuming too many carbohydrates can encourage harmful bacteria and yeast, which exacerbates intestinal dysbiosis [257].

Slow-digesting, fiber-rich carbs that support good gut bacteria and reduce fungal overgrowth should be prioritized over refined carbohydrates and quickly digested starches [257]. Legumes like lentils, chickpeas, and black beans, as well as non-starchy fiber-rich vegetables like broccoli, cauliflower, zucchini, asparagus, leafy greens, and modest amounts of carrots, are some of the suggested substitutes [258]. Other choices include seeds and nuts with prebiotic properties, such as walnuts, flaxseeds, chia seeds, and almonds, or pseudo-cereals with a lower glycemic impact, such as quinoa, buckwheat, and amaranth, when taken in moderation [259,260].

Recently, resistant starch sources that do not quickly raise blood sugar levels are becoming increasingly popular. These include cooked and cooled brown rice (which has a lower glycemic impact than white rice), cooked and cooled potatoes (after refrigeration for 12 to 24 h, as this increases resistant starch content), and unripe (green) bananas, which contain prebiotic fibers that support good gut bacteria [261,262].

Including natural antifungal chemicals in food is one of the complementary methods for antifungal support for the gut flora. These include garlic, which is high in the natural antifungal agent allicin; oregano oil, which contains the antifungal compounds carvacrol and thymol; coconut oil, which contains the antifungal compound caprylic acid; ginger and turmeric, which have anti-inflammatory and antimicrobial properties; and cloves, which contain the antifungal compound eugenol [263,264,265].

**Figure 4:**
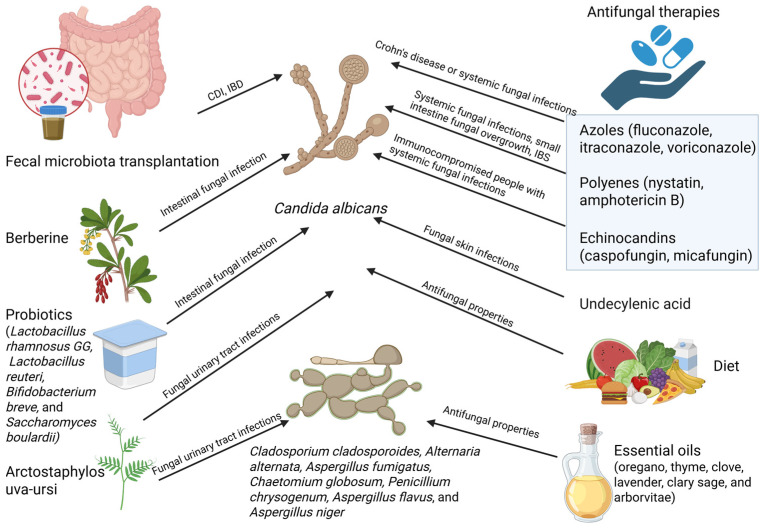
Different methods for calibrating the gut mycobiome. Most approaches target infections caused by *C. albicans*, while some studies suggest that certain methods are effective against both *C. albicans* and other fungal species or exclusively against non-*Candida* fungi. Created with Biorender.com (accessed on 1 March 2025). CDI = *Clostridium difficile* infection; IBD = inflammatory bowel disease.

Polyphenols, which are abundant in foods like cranberries, blueberries, dark chocolate, and green tea, have been demonstrated to promote good bacteria while preventing the growth of fungi [231,266,267]. While alcohol can harm the gut lining and disturb the microbial balance, dairy products, especially those that include lactose, may encourage the growth of *Candida* [244,268,269]. To summarize this subchapter, Figure 4 illustrates various methods for calibrating the gut mycobiome.

## 6. Technological Advances in Mycobiome Research and Future Challenges

Research on the gut mycobiome faces numerous challenges, primarily due to methodological limitations [270]. Culture-dependent techniques, such as biochemical assays, microscopy, and fungal cultivation, remain widely used due to their affordability and cost-effectiveness. Recent advances in metagenomics and the development of non-culture-based methods, such as the polymerase chain reaction (PCR) and high-throughput next-generation sequencing (NGS), have significantly improved our understanding of the gut mycobiome’s composition and function [270]. However, our knowledge remains incomplete due to fungal communities’ high inter- and intra-individual variability [6,9,270].

The targeted amplicon sequencing of the internal transcribed spacer (ITS) regions is now the method of choice for profiling fungal communities. Two main markers are commonly used: ITS1 and ITS2. The ITS1 region offers high variability, allowing for fine-scale resolution at the species level. This advantage is counterbalanced by potential amplification biases, which may skew the observed community composition [271,272]. The ITS2 region, with its more conserved primer binding sites, often yields more consistent amplification across diverse fungal taxa. While ITS2 might provide slightly lower taxonomic resolution compared to ITS1, its consistency is beneficial for cross-study comparisons [273]. Choosing between ITS1 and ITS2 should be guided by the specific objectives of each study, as both have distinct advantages and limitations.

The vast amount of data generated by NGS platforms requires performant bioinformatics tools. One of the key resources in this is the UNITE database, a comprehensive and curated repository of fungal ITS sequences; UNITE improves the accuracy of taxonomic assignments and helps resolve ambiguities, particularly with poorly characterized “dark taxa”. Using tools like UNITE not only improves the reliability of the results but also makes it possible to compare findings across different studies [274,275]. Recent work in clinical mycobiome research further emphasizes that high-quality, curated reference databases like UNITE are indispensable for accurate fungal identification and for advancing diagnostic applications [276].

While the Illuma MiSeq platform has been favored for its high throughput and cot-effectiveness, newer sequencing technologies like PacBio and Oxford Nanopore are gaining attention. These platforms can generate full-length ITS amplicons, which helps in obtaining a more complete and accurate view of fungal communities. Although these technologies face challenges such as higher error rates and cost considerations, ongoing improvements promise to make them highly valuable for future mycobiome research [272]. The benefits of these advanced NGS methods extend well beyond clinical applications. For example, ecological studies have successfully applied these techniques to uncover the diversity of wood-decaying fungi in neotropical Atlantic forests. Such work highlights the versatility of NGS technologies, which are essential for understanding fungal roles in both human health and natural ecosystems [277]. By combining cutting-edge sequencing technologies with comprehensive reference databases like UNITE, researchers are paving the way for more targeted microbiome-based therapies and a deeper understanding of fungal contributions to various biological processes.

Additionally, technical challenges and host-related factors—including age, diet, immunity, and genetics—may contribute to underestimating the gut mycobiome’s significance [270,278]. Although previous reviews have extensively covered fungus–disease associations and interventions, our review distinguishes itself by incorporating recent high-impact studies. A recent landmark study involving a cohort of 12,641 Chinese individuals, using internal transcribed spacer (ITS) sequencing, revealed novel associations between specific fungal taxa and host metabolic health [279]. The findings highlight the role of fungal alpha diversity and 19 mycobiome genera in cardiometabolic diseases, as well as a notable link between *Saccharomyces* and type 2 diabetes. In parallel, enhanced insights into the metabolic crosstalk between gut bacteria and fungi—reviewed in part by Zhang et al., MacAlpine et al., and Deveau et al.—suggest that fungal dysbiosis may be a causative factor in disease progression rather than a secondary phenomenon [4,42,74]. Furthermore, recent advances in the understanding of immune regulation have identified specific fungal components that shape T-cell responses and modulate inflammation, thereby providing a mechanistic basis for targeted dietary, probiotic, and FMT interventions [41,125]. Future research must clarify the precise role of fungi in disease development—whether fungal dysbiosis is a cause or consequence of illness [4]. For instance, increased *C. albicans* levels in patients with CDI, IBD, or COVID-19 could either disrupt gut microbial equilibrium (causative factor) or result from antibiotic use (consequence) [12,24,48]. It is also essential to distinguish whether a disease is caused by pathogenic fungi from external sources (e.g., food) or by commensal fungi residing in the gut, as this distinction will directly impact treatment strategies [270]. Further studies must explore how fungal inter- and intra-individual variability affects disease progression and therapeutic responses, paving the way for targeted gut mycobiome-based therapies [24]. Additionally, future research should investigate the interactions between the gut mycobiome and other microbial communities (bacteria, viruses, and archaea) and their influence on host-specific factors such as immunity, ethnicity, and diet [280,281]. Understanding these interactions is crucial, especially given that the gut bacteriome and virome exhibit high variability across individuals [282]. Moreover, the relationship between the gut mycobiome and host metabolic pathways remains an emerging area of interest. Notably, *Candida* species and the gut–brain axis interplay have been implicated in disease development [283]. Encouragingly, fungal-based therapies show potential; for example, *Saccharomyces boulardii* supplementation improved intestinal neuromuscular anomalies in mice with IBD, while *Candida kefyr* administration ameliorated autoimmune encephalomyelitis in a multiple sclerosis model [284,285]. These findings highlight the therapeutic potential of gut mycobiome modulation and underscore the need for further research to harness fungal communities for disease prevention and treatment.

## 7. Conclusions

The gut mycobiome is an emerging and complex component of the human microbiome, playing a crucial role in maintaining health and contributing to disease pathogenesis. While recent studies have significantly advanced our understanding of its composition, interactions, and clinical relevance, many challenges remain. The balance between fungi and bacteria is essential for gut homeostasis, and disruptions in this equilibrium have been associated with conditions such as IBD, metabolic disorders, and infections. Despite advances in sequencing technologies and metagenomics, research on the gut mycobiome is still in its infancy, hindered by methodological limitations, high inter-individual variability, and the complex interplay between fungi, bacteria, and host factors. Clinical interventions targeting the gut mycobiome, including antifungal therapies, probiotics, FMT, and dietary modifications, show promising potential but require further validation through large-scale, well-controlled studies. Future research should focus on defining the precise role of gut fungi in disease progression, improving diagnostic methods, and developing targeted therapeutic strategies. Additionally, understanding how fungal communities influence host immune responses and metabolic pathways will be crucial for designing novel microbiome-based treatments. As research in this field expands, integrating fungal microbiome data with bacterial and viral microbiota studies will provide a more comprehensive view of human gut health and disease, ultimately paving the way for personalized microbiome-based medicine.

## Figures and Tables

**Table 1:** The most commonly identified fungi genera and their effect on human health and disease.

Study	Fungi	Disease	Effect	Ref.
Pfaller et al.	*C. albicans*	Invasive fungal infection	Exacerbates	[46]
Li et al.	*C. albicans*	Antibiotic-associated diarrhea	Exacerbates	[47]
Charlet et al.	*C. glabrata*	Colitis	Exacerbates	[51]
Martino et al.	*C. tropicalis*	Colitis	Exacerbates	[52]
Hoarau et al.	*C. tropicalis*	CD	Exacerbates	[53]
Sun et al.	*C. parapsilosis*	Diet-related obesity	Exacerbates	[54]
Sokol et al.	*C. albicans*	CD and UC	Exacerbates	[48]
Chester et al.	*Clavispora lusitaniae* (formerly *Candida lusitaniae*)	Digestive and urinary infections Amphotericin B resistance	Induces	[55]
Noor-UI et al.	*Geotrichum candidum*		Probiotic that enhances feed utilization, improves immunity, and reduces disease resistance	[56]
Abid et al.	*Saccharomyces*	Traveler’s diarrhea and CDI	Protects	[58]
Chen et al.	*Saccharomyces*	Colon cancer	Antineoplastic effects	[60]
Spatz et al.	*Malassezia*	Dermatitis and pityriasis	Exacerbates	[13]
Limon et al.	*Malassezia*	Inflammatory bowel disease	Exacerbates	[15]
Sokol et al.	*Malassezia*	Inflammatory bowel disease	Exacerbates	[48]
Yang et al.	*Malassezia*	CRC	Unclear—leads to tumorigenesis	[68]
Norlia et al.	*Aspergillus*	Hepatocellular carcinoma	Hepatotoxic effect	[71]
Malir et al.	*Penicillium*	Urothelial carcinoma	Nephrotoxic effect	[72]

CD = Crohn’s disease; UC = ulcerative colitis; CRC = colorectal cancer; *C. albicans* = *Candida albicans*; *C. glabrata* = *Candida glabrata* ; *C. tropicalis* = Candida tropicalis; *C. parapsilosis* = *Candida parapsilosis*.

## Data Availability

No new data were created or analyzed in this study.

## Abbreviations

The following abbreviations are used in this manuscript:

AhR	Aryl hydrocarbon receptor
CD	Crohn’s disease
CDI	*Clostridium difficile* infection
CRC	Colorectal cancer
FMT	Fecal microbiota transplantation
IBD	Inflammatory bowel disease
MS	Multiple sclerosis
RA	Rheumatoid arthritis
SCFAs	Short-chain fatty acids
SIFO	Small intestine fungal overgrowth
T2D	Type 2 diabetes
Tregs	Regulatory T cells
UC	Ulcerative colitis
